# The role of inflammatory factors in the tumor microenvironment of pancreatic cancer

**DOI:** 10.3389/fimmu.2025.1625114

**Published:** 2025-08-28

**Authors:** Yuzhang Yuan, Haozhe Zhang, Zehua Wang, Luying Huang, Derya Kabacaoglu, Boxing Zhang, Liang Song, Jiaoyu Ai

**Affiliations:** ^1^ Department of Oncology, The First Affiliated Hospital of Zhengzhou University, Zhengzhou, China; ^2^ Department of Gastroenterology, The First Affiliated Hospital of Nanchang University, Nanchang, China; ^3^ Comprehensive Cancer Center Munich CCCM, Technical University of Munich, Munich, Germany; ^4^ Medical Experiment Center, Shaanxi University of Chinese Medicine, Xianyang, China

**Keywords:** pancreatic cancer, inflammatory factors, tumor microenvironment, immunology, targeted therapy

## Abstract

Pancreatic cancer (PC) is an aggressive malignancy with a poor prognosis, and the tumor microenvironment (TME) plays a pivotal role in its initiation, progression, and response to treatment. Recent studies have highlighted the critical involvement of inflammatory factors in shaping and sustaining the PC microenvironment. Chronic inflammation is a hallmark of this cancer, with inflammatory molecules such as cytokines, chemokines, proteases, and other immune-modulatory factors driving tumor cell proliferation, metastasis, and resistance to therapy. These inflammatory factors exert their effects by modulating immune cell infiltration, extracellular matrix (ECM) remodeling, and angiogenesis. This review provides an overview of the diverse roles of inflammatory factors in the PC TME and explores their potential as therapeutic targets. It offers new perspectives for developing novel immunotherapies and inflammation-modulating strategies to improve the treatment of PC.

## 1 Introduction

PC represents a growing public health concern, currently ranking as the third-leading cause of cancer-related mortality in the United States, with an annual increase in mortality of 0.3% since 2000 (1). Projections suggest it will become the second-leading cause of cancer-related deaths by 2030 (2). The prognosis for PC remains dismal, primarily due to late-stage diagnosis, as the majority of patients are diagnosed after the disease has already metastasized (3–6). Furthermore, the limited effectiveness of current treatment options, coupled with inherent resistance to standard therapies, contributes to a 5-year survival rate of 13.3% (7, 8). Given these challenges, it is imperative to further investigate the pathogenesis of PC and develop novel therapeutic strategies.

The profound influence of the TME on tumor progression has been well-documented in numerous studies. PC, however, exhibits distinct TME characteristics that differentiate it from other malignancies. In the PC TME, T cell dysfunction, driven by a variety of mechanisms, plays a pivotal role in tumor progression and is a central contributor to the immunosuppressive milieu that characterizes this cancer (9). Moreover, the presence of extensive fibrosis and the resulting low perfusion further complicate the development of effective therapies targeting the microenvironment, presenting an additional challenge in the treatment of PC (10).

Inflammatory factors play a pivotal role in the aforementioned alterations of the TME. Inflammation is a complex physiological process initiated by the immune system, wherein immune cells are mobilized to respond to signals such as wounds, infections, or other irritants. Upon activation, these immune cells release a variety of chemical mediators that recruit additional specialized immune cells to the site of injury, thereby driving the inflammatory response. This accumulation of immune cells and the release of pro-inflammatory molecules characterize the inflammatory process. Previous studies have established a strong correlation between pancreatic inflammation and the onset and progression of PC (11), with inflammatory factors playing a central role in this association (12). In one respect, inflammation promotes the survival and proliferation of cancer cells through the secretion of inflammatory mediators such as cytokines. In another respect, inflammatory cytokines serve as key modulators of TME changes, thus contributing to tumor progression. Given the significant role of inflammation in the development and progression of PC, targeting inflammatory cytokines has emerged as a promising therapeutic strategy. This review aims to provide an overview of the mechanisms by which inflammatory factors influence the TME and, consequently, tumor progression in PC, while also discussing the feasibility and potential of existing therapeutic approaches that target these inflammatory pathways.

In light of the aforementioned background, we have endeavored to summarize the current clinical trials targeting inflammation-mediated alterations in the TME, as well as the potential therapeutic targets for PC. The ultimate aim of this effort is to enhance the prognosis of patients with PC.

## 2 The key features of the TME in PC

The tumorigenesis of PC is a complex, multistep process (13). The most prevalent form of PC is pancreatic ductal adenocarcinoma (PDAC), which accounts for approximately 85% of all pancreatic malignancies (14). The onset of cancer involves two primary types of changes: genetic mutations in the pancreatic ductal cells and alterations in the composition and function of the pancreatic stroma. Pancreatic intraepithelial neoplasia (PanIN) is considered the most likely precursor lesion of PDAC, characterized by preneoplastic mucinous lesions with ductal morphology (15). KRAS mutations play a pivotal role in the initiation of this process (16), while mutations in other genes such as TP53, CDKN2A, and SMAD4 also contribute significantly, working in concert with KRAS mutations to drive the progression of PDAC (17, 18). In addition to the mutations within the pancreatic ductal cells, the influence of stromal changes is equally critical, as various alterations in the stromal microenvironment play an indispensable role in the development of PC.

### 2.1 Immunosuppressive TME

**Figure 1:**
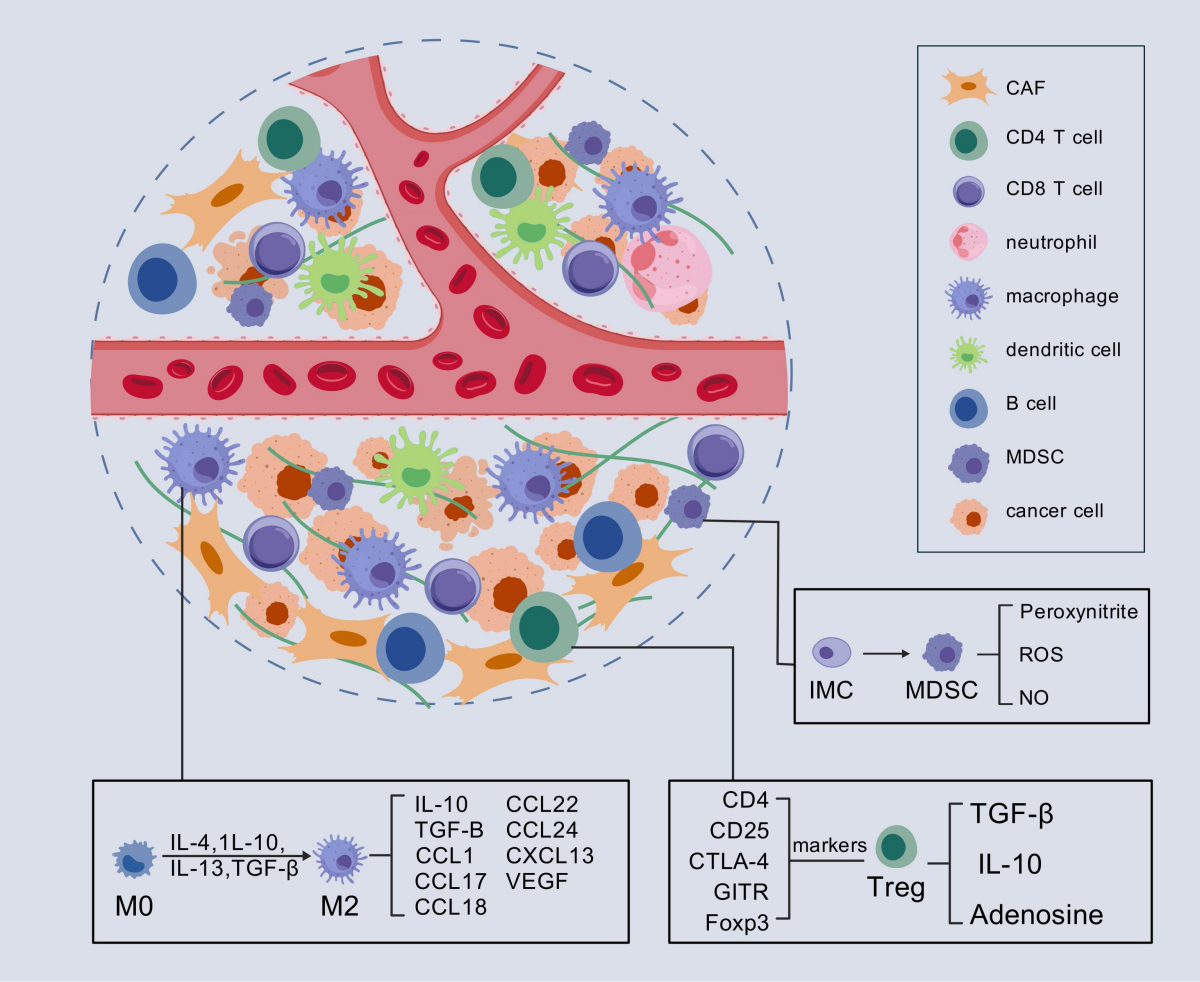
Immunosuppressive TME in PC. In the immunosuppressive TME of PC, Tregs, TAMs with M2 polarization, and MDSCs play key roles. They alter signaling pathways within the TME by secreting various inflammatory factors, which in turn lead to the suppression of T cell function. CAF, cancer-associated fibroblasts; IMC, immature myeloid cell; MDSC, myeloid-derived suppressor cell; Treg, regulatory T cells. ROS, reactive oxygen species.

Compared to other cancers with high mutational burdens, PC exhibits a relatively low mutational burden, resulting in a limited number of potential targets for immune recognition (19). Consequently, immunotherapy through checkpoint inhibition has yielded only modest success in patients with PDAC, in contrast to its more substantial benefits in other malignancies. Research has shown that the TME of many PDACs is characterized by an increased prevalence of immunosuppressive cell types, including T-regulatory cells (Tregs), tumor-associated macrophages (TAMs) with M2 polarization, and myeloid-derived suppressor cells (MDSCs), all of which contribute significantly to the immunosuppressive milieu (20–22). The accumulation of these immunosuppressive cells correlates with enhanced malignancy and aggressiveness of PC (23). These cells primarily inhibit antitumor immunity by reducing both the number and the functional activity of effector T cells (**Figure 1**). Oncogenic KRAS-induced upregulation of granulocyte-macrophage colony-stimulating factor (GM-CSF) fosters the recruitment of GR1(+)CD11b(+) MDSCs, a process that is mediated by CD8(+) T cells. Consequently, the presence of GR1(+)CD11b(+) myeloid cells suppresses the proliferation of CD8+ T cells (24). Additionally, Treg cells have been shown to inhibit the production of costimulatory ligands necessary for the activation of CD8+ T cells by restricting the immunogenic functions of tumor-associated CD11c+ dendritic cells (DCs), thereby limiting CD8+ T cell responses (25). Moreover, TAMs play a crucial role in PC tumorigenesis. An inflammatory feedback loop has been identified between TAMs expressing interleukin-1β (IL-1β) and tumor cells, which serves as a precursor to pancreatic carcinogenesis (26). Further studies have demonstrated that TAMs contribute to pancreatic acinar-ductal metaplasia through the secretion of tumor necrosis factor (TNF), RANTES, and the induction of matrix metalloproteinase 9 (MMP9) (27). These findings indicate that the immunosuppressive effects of these cell populations are largely mediated by inflammatory cytokines and their receptors, thus offering valuable insights for the identification of novel therapeutic targets in the treatment of PC.

### 2.2 Dense desmoplastic stroma and cancer-associated fibroblasts

The TME of PC is characterized by extensive fibrosis, with stromal components constituting the majority of its volume (28). Key ECM components, such as collagen and hyaluronan (HA), are prominently involved, and elevated levels of HA in PDAC are strongly associated with poor prognosis (29, 30). CAFs play a significant role in the fibrosis process. The sources of CAFs in PDAC are extensive, such as resident fibroblasts, mesenchymal stem cells (MSCs), tissue-resident fibroblasts, and pancreatic stellate cells (PSCs) (31–33). These CAFs are functionally classified into three major subtypes: myofibroblastic CAFs (myCAFs) that promote extracellular matrix stiffening and create mechanical tumor barriers, inflammatory CAFs (iCAFs) which secrete cytokines to establish an immunosuppressive microenvironment, and antigen-presenting CAFs (apCAFs). Importantly, these functionally distinct CAF subtypes demonstrate plasticity and can interconvert under specific microenvironmental conditions, as evidenced by recent single-cell and spatial transcriptomic studies (34). CAFs play a crucial role in promoting fibrosis within the TME. For instance, the knockout of NID2 has been shown to suppress CAF activation, thereby inhibiting both tumor fibrosis and metastasis, while concurrently modulating tumor vasculature and enhancing therapeutic efficacy (35). Traditionally, fibrosis in tumors has been understood through the perspective that the ECM acts as a physical barrier that obstructs drug delivery, thereby reducing the efficacy of therapeutic interventions. However, recent insights suggest a more complex interaction wherein CAFs, in conjunction with their native ECM, function as an integrated entity (36). This CAF-ECM complex not only serves as a physical barrier but also acts as a reservoir for secretory factors, which play a pivotal role in tumor progression (37, 38).

Moreover, CAFs can influence tumor growth by modulating key metabolic pathways, including lipid, amino acid, and polyamine metabolism (39–41). Among these, lipid metabolism has been particularly well-explored. A recent study demonstrated that the loss of Setd2 results in an aberrant increase of H3K27Ac at the gene body of Bmp2, which subsequently drives the differentiation of adjacent CAFs into a lipid-enriched phenotype. These lipid-laden CAFs, in turn, supply lipids to Setd2-deficient tumor cells, thereby supporting mitochondrial oxidative phosphorylation (OXPHOS) and promoting tumor growth (42).

Several therapeutic strategies aimed at targeting CAFs or disrupting their interactions with other immune cells have demonstrated varying degrees of success in both preclinical studies and clinical trials (NCT01130142 and NCT03563248). The ECM acts as both a physical and metabolic barrier to drug delivery, contributing to the resistance of PDAC to chemotherapy, targeted therapies, and even immunotherapy (43). Recent research has highlighted that targeting CAFs’ autophagic processes can enhance the efficacy of immunochemotherapy in PC by alleviating adaptive immune resistance. This approach holds promise as a potential novel therapeutic target for the treatment of PC in the future (44).

### 2.3 Hypoperfusion

To ensure an adequate supply of nutrients and support tumor metastasis, most tumors secrete a variety of pro-angiogenic factors to promote angiogenesis (45). In PC, the interstitial fluid pressure (IFP) is significantly elevated compared to other cancers, primarily due to a pronounced fibroinflammatory response. This elevated IFP leads to hypoperfusion within the tumor, resulting in poor delivery of small molecule drugs and the creation of a hypoxic TME (46). The former contributes to drug resistance, while the latter induces the overexpression of hypoxia-inducible factor (HIF)-1α in PC cells. HIF-1 is a key regulator of the cellular response to hypoxia, and the expression of HIF-1α is closely associated with tumor progression and metastasis (47). HIF-1α functions by directly binding to hypoxia-response elements (HREs) in the promoters of target genes, activating downstream pathways that enable cells to adapt to low oxygen conditions. However, recent perspectives suggest that the regulation of HIF-1α in tumors is more complex than merely promoting tumor development. As such, rather than targeting HIF-1α directly, addressing the hypoxic TME itself may offer more effective therapeutic outcomes in PC (48, 49).

**Figure 2:**
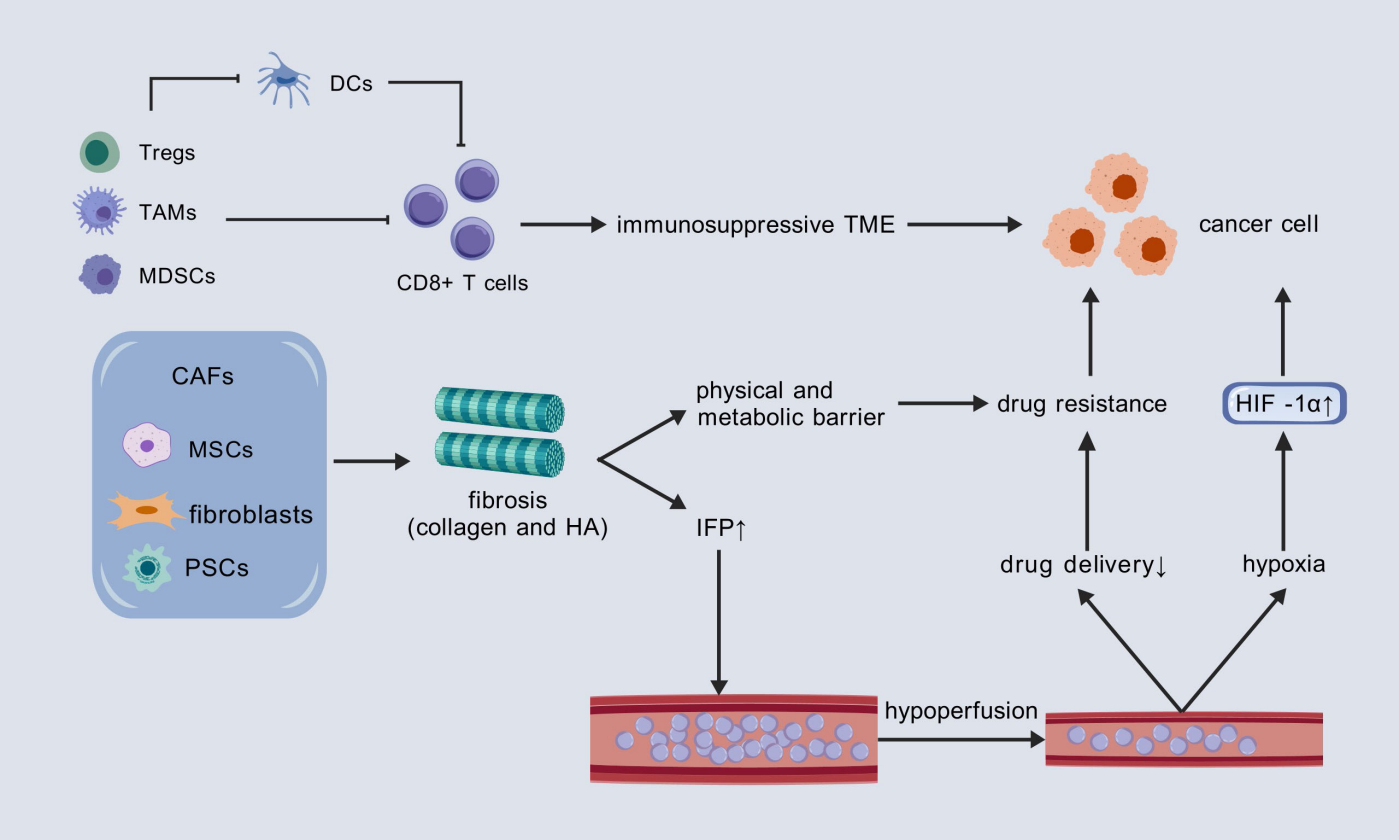
Three core mechanisms promoting tumor progression in the PC TME and their interconnections. DCs, dendritic cells; TAM, tumor-associated macrophage; MDSC, myeloid-derived suppressor cell; CAF, cancer-associated fibroblast; MSC, mesenchymal stem cell; PSCs, pancreatic stellate cells; HA, hyaluronic acid; IFP, interstitial fluid pressure; HIF-1α, hypoxia-inducible factor 1-alpha.

Notably, these characteristics may be interrelated and potentially causative, rather than entirely independent from one another (**Figure 2**). For instance, stromal fibrosis can physically obstruct the infiltration of immune cells and encapsulate tumor tissue, thereby contributing to the formation of a hypoxic microenvironment. Hypoxia, in turn, activates Lactate dehydrogenase A (LDHA), which subsequently promotes the production and secretion of L-2HG. This cascade of events ultimately results in the inhibition of T cell proliferation and migration (50).

## 3 Inflammatory factors’ impact on PC TME

**Figure 3:**
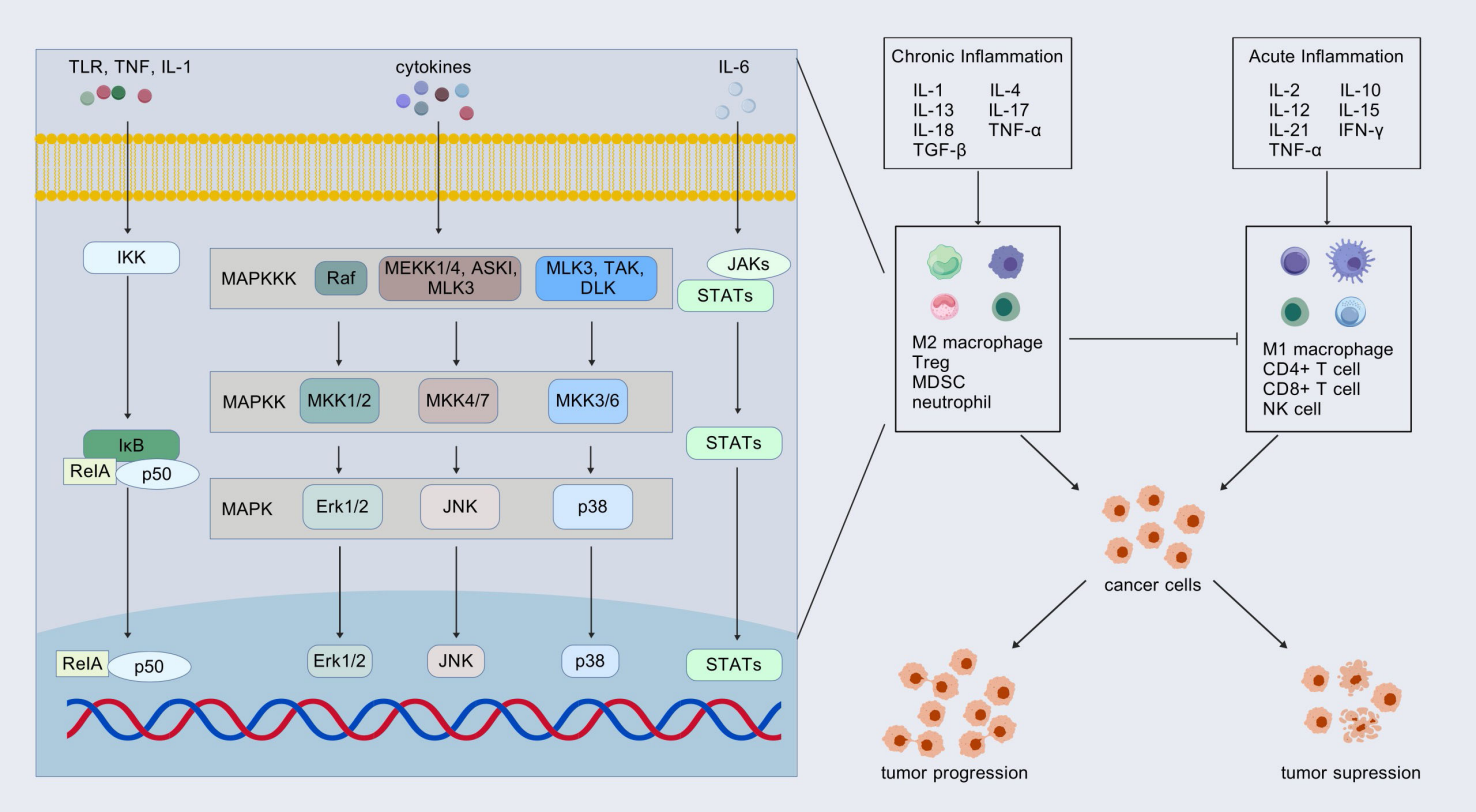
Inflammatory factors: friends or foes? Inflammatory factors in acute and chronic inflammation play opposite roles in tumor progression by affecting the TME. These inflammatory factors transfer information into the cell through signaling pathways. The most common pathways involved include mitogen-activated protein kinases (MAPK), Janus kinase (JAK)-STAT, and NF-κB pathways. Treg, regulatory T cell; MDSC, myeloid-derived suppressor cell.

Inflammatory factors serve as a crucial link between the TME and tumor cells through complex regulatory mechanisms. Acute inflammation typically promotes the immune response, whereas chronic inflammation may lead to immunosuppression. In the context of chronic inflammation, several inflammatory mediators, including IL-6, IL-1, IL-17A, IL-22, and transforming growth factor beta (TGF-β), have been shown to exert pro-tumorigenic effects. These factors, which influence cell fate, can be hijacked by mutated cells, thereby activating key signaling pathways such as mitogen-activated protein kinases (MAPK), phosphatidylinositol-3-kinase (PI3K)-Akt, Janus kinase (JAK)-STAT, and NF-κB, thereby increasing the risk of tumorigenesis (51, 52). Inflammatory mediators play a significant role in the early malignant progression of various cancers, including esophageal squamous cell carcinoma, hepatocellular carcinoma, and intrahepatic cholangiocarcinoma (53). Building upon existing research, we review the inflammatory factors that contribute to the initiation and progression of PC through their influence on the TME (**Figure 3**).

### 3.1 Interleukin-1β

Among these cytokines, IL-1β stands out as a key player in PC pathogenesis. IL-1β, a factor secreted by various cells, has pleiotropic effects on immune cells, angiogenesis, cancer cell proliferation, migration and metastasis. In the context of PC, tumor cell expression of IL-1β *in vivo* was driven by microbial-dependent activation of Toll-like receptor 4 (TLR4) signaling and subsequent engagement of the NLRP3 inflammasome. As a cancer-promoting factor, IL-1β promotes tumor immune escape by interacting with stromal cells (54). As a bridge, it connects various stromal cells such as fibroblasts, macrophages, stellate cells and PC cells, and plays an important role in the occurrence and development of PC. As a cancer-promoting factor, IL-1β promotes tumor immune escape by interacting with stromal cells. As a bridge, it connects various stromal cells such as fibroblasts, macrophages, stellate cells and PC cells, and plays an important role in the occurrence and development of PC. Recent studies have demonstrated that tumor-infiltrating monocytes differentiate into IL-1β-producing TAMs following exposure to prostaglandin E2 (PGE_2_) and TNF. These IL-1β+ TAMs subsequently interact with IL-1β-reactive PC cells, thereby promoting tumor progression. This process highlights the PGE_2_–IL-1β axis as a critical driver of spatial and transcriptional heterogeneity within both immune and tumor cells in PDAC. Furthermore, inflamed PDAC tissue and TAMs engage in a positive feedback loop that exacerbates cancer progression (26). In addition to macrophages, a similar positive feedback pathway is observed in PSCs. ESE3, a transcription factor, induces the transcription of α-SMA, collagen I, and IL-1β by binding to specific ESE3 response elements on their promoters. As a result, IL-1β upregulates ESE3 expression in PSCs through NF-κB activation, and ESE3 is essential for PSC activation by tumor-derived IL-1β (55). Furthermore, technological advances in single-cell and spatial transcriptomics have enabled precise characterization of the role and mechanisms of IL-1β within tumor tissues. IL-1β exhibits non-uniform spatial distribution in tumors. These studies revealed that IL-1β^+^ TAMs preferentially localize to hypoxic stromal regions and directly neighbor tumor cells expressing the IL-1β Response Signature (TIRS). Critically, IL-1β^+^ TAMs activate adjacent TIRS^+^ PDAC cells through IL-1β secretion, triggering the release of PGE_2_ and TNF. These factors then synergistically drive monocyte differentiation into IL-1β^+^ TAMs, establishing a self-amplifying circuit that perpetuates tumor progression (26).

### 3.2 Interleukin-4

Building on the role of IL-1β, IL-4 also exerts a profound influence on PC. Significantly elevated levels of IL-4 have been observed in cancer cases compared to control participants (56). Interleukin-4 (IL-4) exerts a dual effect in human PC, acting both directly on the tumor cells themselves and indirectly within the TME. PC cells serve as both a source and target of IL-4, creating an autocrine loop that directly promotes tumor cell proliferation. This pro-proliferative effect can be counteracted by neutralizing antibodies against IL-4 (57). Beyond its autocrine action, IL-4 acts paracrinely to establish an immunosuppressive TME. A key mechanism involves IL-4 inducing the polarization of macrophages towards an immunosuppressive M2 phenotype (58). In addition to shaping macrophage function, IL-4 directly impairs anti-tumor immunity by inhibiting T cell effector functions. IL-4 secreted within the TME binds to the IL-4 receptor on T cell surfaces, suppressing their activity and facilitating tumor immune escape. Consequently, strategies aimed at blocking IL-4 signaling within this immunosuppressive TME, such as engineering CAR-T cells to co-express IL-4/IL-15-based inverted cytokine receptors, represent promising therapeutic approaches. This strategy could enhance immunotherapy efficacy, particularly in tumors like PC characterized by elevated IL-4 levels (59).

### 3.3 Interleukin-13

IL-13 follows a similar pattern of dysregulation in PC. IL-13 is expressed at significantly higher levels in PC compared to normal tissues (60), and its elevated expression is considered an unfavorable prognostic factor for PC (61). The primary receptor subunits for IL-13 are IL-13Rα1 and IL-13Rα2, both of which are involved in promoting the progression of PC by acting on cancer cells (61, 62). Notably, while IL-13Rα1 is ubiquitously expressed in healthy tissues, IL-13Rα2 exhibits tumor-specific overexpression with minimal distribution in normal organs, positioning it as a high-value target for chimeric antigen receptor (CAR) T-cell therapy in PDAC. Engineered IL-13 mutein-based CARs have thus been developed to exploit this selectivity for PDAC treatment. Compared to conventional single-chain variable fragment (scFv)-based CARs, these ligand-directed designs not only mitigate immunogenicity risks (63) but also offer potential multi-tumor targeting capabilities with reduced engineering complexity (64). Within the TME, IL-13 serves as a key mediator bridging PSCs and macrophages. PSCs, which are a major source of IL-4 and IL-13 (65), contribute to the tumorigenic process. In lesions such as acinar-to-ductal metaplasia (ADM) and panIN, IL-13 induces the polarization of inflammatory macrophages into Ym1+ alternatively activated macrophages. These Ym1+ macrophages, in turn, secrete factors such as CCL2 and IL-1 receptor antagonist (IL-1Ra), which further support tumorigenesis and pancreatic fibrogenesis (66).

### 3.4 Interleukin-15

Shifting focus to another cytokine, IL-15 predominantly stimulates the proliferation and cytotoxic activity of CD8+ T cells and NK cells, enhancing antitumor immune responses. Although research on the role of IL-15 in PC remains limited, recent studies have highlighted its potential as a mediator in exercise-induced tumor immunity. Kurz et al. demonstrated that aerobic exercise inhibited PC growth in mice by activating the immune system, particularly through the activation of CD8+ T cells. Moreover, Niz985, an IL-15 super-agonist, was shown to replicate the beneficial effects of exercise on tumor immunity. Notably, both Niz985 and exercise significantly enhanced the therapeutic sensitivity of otherwise intractable pancreatic tumors in mice. This pioneering study revealed that exercise promotes antitumor immunity in PC via activation of the IL-15/IL-15Rα signaling axis, suggesting that this pathway may represent a promising therapeutic strategy (67). As a central regulator of NK cell survival, proliferation, and cytotoxicity, IL-15 expression in CAFs is suppressed by nociceptor neuron-derived CGRP. Consequently, NK cell infiltration and activation are inhibited. Multiplex immunofluorescence of PDAC tissues showed reduced CAF-derived IL-15 and diminished NK cell presence in regions with high CGRP^+^ nerve density (e.g., tumor periphery or perineural invasion sites). Spatial analysis confirmed a significant inverse correlation between CGRP^+^ nerve density and IL-15/NK cell levels, indicating concentrated IL-15 suppression in nerve-rich regions (68).

### 3.5 Interleukin-17

IL-17 also plays a crucial role in PC initiation and progression by regulating CAFs, T cells, and neutrophils. Primarily secreted by CD4+ and γδ T cells, IL-17 promotes the formation of panIN lesions (69, 70). In the context of PC, IL-17 contributes to the maintenance of immunosuppression by decreasing the recruitment of CD8+ T cells while simultaneously increasing the infiltration of neutrophils into the TME. Furthermore, IL-17 triggers the formation of neutrophil NETs, which leads to the remodeling of the ECM and surrounding tissue. This process facilitates the development, spread, and metastasis of the cancer (71). IL-17 also exerts significant regulatory effects on CAFs. Specifically, Tc17 cells, a subset of CD8+ T cells that produce IL-17A, promote the transformation of IL-17RA+ CAFs into an inflammatory phenotype through the secretion of IL-17A and TNF. This, in turn, fosters the progression of PDAC by altering the transcriptome of PC cells, thereby enhancing tumor cell proliferation (72). In addition, a recent study has revealed that IL-17 may promote tumor progression by inhibiting fibrosis in CAFs, which challenges the conventional view that fibrosis within the TME enhances PC progression. This finding underscores the need for a more nuanced classification of the various components within the TME, which could aid in refining therapeutic strategies and improving treatment precision (73). Moreover, IL-17 interacts with a variety of other cytokines, further complicating its role in tumor biology. For instance, the IL-17B/IL-17RB axis activates the expression of chemokines such as CCL20, CXCL1, IL-8, and TFF1 via the ERK1/2 signaling pathway, which in turn influences tumor metastasis and the recruitment of macrophages and endothelial cells. This highlights the critical involvement of IL-17 in the TME and its potential as a therapeutic target to enhance treatment efficacy (74). Beyond its role within the TME, circulating IL-17 has also been implicated in the carcinogenesis and metastasis of PC, further emphasizing the complexity of IL-17’s functions in cancer biology (75).

### 3.6 Interleukin-18

IL-18, recognized as a pro-cancer factor, is strongly associated with poor prognosis in patients (76). Similar to other cytokines previously studied, IL-18 plays a significant regulatory role in macrophages and CD8+ T cells within the PC microenvironment, thereby contributing to tumor progression. Recent studies have identified IL-18 as a key downstream mediator of GFPT2, wherein GFPT2-mediated O-GlcNAcylation of YBX1 enhances its nuclear translocation and promotes the transcription of IL-18. This process is critical for M2 macrophage polarization in PC (77). Furthermore, research into T cell exhaustion has highlighted the crucial role of IL-18 receptor (IL-18R) signaling in this phenomenon. It has been demonstrated that IL-18, acting as a downstream molecule of NLRP3, activates both the IL-2/STAT5 and AKT/mTOR pathways upon binding to IL-18R. This signaling cascade promotes CD8+ T cell exhaustion, contributing to immune evasion in tumors (76). In addition to these immune-suppressive mechanisms, other studies have shown that IL-18 can drive eosinophil accumulation, leading to eosinophilic chronic inflammation. This inflammation promotes pancreatic tissue remodeling and fibrosis, which is closely linked to the initiation and progression of pancreatic ADM and panIN. These processes may represent the critical early steps in the transition from chronic pancreatitis (CP) to PC (78).

### 3.7 Tumor necrosis factor

Finally, TNF-α has been extensively studied for its pivotal role in PC initiation and progression (79, 80). TNF-α is predominantly secreted by macrophages and exerts its biological effects through binding to two distinct receptors: the death-domain-containing TNF receptor 1 (TNFR1) and the tissue-restricted TNF receptor 2 (TNFR2) on the cell surface (81–83). TNFR1 is broadly expressed across various cell types, including tumor cells and CAFs, and it mediates pro-apoptotic pathways through the activation of its death domain. In contrast, TNFR2 is primarily expressed on immune cells and endothelial cells, and its signaling promotes the activation of NF-κB through a non-classical pathway, without inducing cell death. Both of these pathways are implicated in the pathogenesis of PC. Some studies have indicated that TNF-α, through its interaction with TNFR1, can inhibit the infiltration and activation of antigen-presenting DCs. Blockade of TNFR1 has been shown to attenuate this inhibitory effect, thereby restoring T cell-mediated anti-tumor immunity and effectively impeding tumor progression (84). On the other hand, with respect to the major microenvironmental characteristics associated with resistance in PC—specifically, the inflammatory polarization of CAFs and T cell dysfunction—recent research has highlighted the significant role of neutrophil-derived transmembrane TNF-TNFR2 interactions in these processes (85). Furthermore, a separate study has suggested that the inhibition of TNF-α expression can lead to an upregulation of IL-33 in tumor cells, which in turn enhances the activity of DCs and cytotoxic T cells, thereby promoting anti-tumor immunity. This may represent one of the downstream mechanisms through which TNF-α exerts its immunosuppressive effects (81). Taken together, these findings indicate that targeting TNF-α or specifically the pro-tumor immunosuppressive TAMs that produce TNF-α may represent a promising therapeutic strategy to counteract the immunosuppressive microenvironment and improve treatment outcomes in PC. Spatial multi-omics (IMC/transcriptomics) reveal TNF operates via direct cell-contact: KRAS-TP53 mutant tumor islands autonomously secrete CXCL1, recruiting CXCR2^+^ neutrophils (PMN-MDSCs) into close proximity (<10 μm). This interaction excludes CD8^+^ T cells (>95 μm), creating immunosuppressive niches. Mechanistically, tmTNF^+^ neutrophils engage TNFR2 on tumor cells, inducing reciprocal CXCL1/IL-6 upregulation (feedforward loop) and driving iCAF differentiation. Resultant iCAFs promote fibrosis and activate therapy-resistant IL-6/STAT3 signaling (86).

### 3.8 Prostaglandins

PGs are a class of lipid mediators derived from arachidonic acid via cyclooxygenase (COX) catalysis, with major subtypes including PGE_2_, PGD_2_, and thromboxane. In PC, upregulation of the cyclooxygenase-2 (COX-2) pathway is prevalent, and its carcinogenic effects are largely attributed to the overproduction of PGE_2_ (87–89). Prospective studies provide further evidence supporting the involvement of the COX-2 pathway in pancreatic carcinogenesis and suggest that urinary prostaglandin E metabolite (PGE-M) may serve as a biomarker for predicting PC risk (90). It is noteworthy that PC cells undergoing endoplasmic reticulum (ER) stress release PGE_2_, which can transfer this stress signal to DCs, impairing their immune function and contributing to immunosuppression (91). Simultaneously, the role of PGE_2_ on CAFs within the TME is crucial. On one hand, PGE_2_ acts on cancer cells to induce their secretion of fibroblast growth factor 1 (FGF1), which subsequently stimulates CAF proliferation and enhances their fibrotic activity. On the other hand, activated CAFs increase the expression of vascular endothelial growth factor A (VEGFA), promoting angiogenesis. These processes collectively drive the formation of a fibrotic TME, not only promoting the initiation and progression of pancreatic tumor tissue but also creating a physical barrier through increased collagen secretion that impedes chemotherapeutic drug delivery (92, 93). Expanding the focus to therapeutic applications, the interaction between PGE_2_ signaling and CAR-T therapy effectiveness is a subject of ongoing research, despite the well-characterized role of PGE_2_ in PC pathogenesis. Existing evidence demonstrates that PGE_2_ significantly suppresses T cell (including CAR-T cell) proliferation and impairs their antitumor function through EP2/EP4 receptor signaling. This underscores the potential value of targeting the PGE_2_ signaling pathway as a strategy to enhance CAR-T cell efficacy and improve therapeutic responses in PC (94).

## 4 The clinical value of inflammatory factors in PC

In recent years, research on inflammatory factors in PC has accumulated certain findings in terms of prognosis evaluation, clinical treatment, and new drug development (**Table 1**).

**Table 1:** The role of inflammatory factors in PC therapy.

Category	Therapy	Inflammatory factors	Mechanism	Ref.
Nanotechnology - based treatment approaches	GEP	TGF-β1	GEP can co-deliver Gp and PFD into PC cells and release them synchronously, overcoming the obstacles posed by their non-specific accumulation and pharmacokinetic differences.	(100)
	SB525334	TGF-β1	SB blocks TGF - induced Smad activation, reduces EMT, specifically attenuates the activation of α - SMA+/FAPα+ myCAFs, improves the TME, and facilitates the delivery of the nanodrug docetaxel micelles.	(102)
Immunotherapy	CTLA-4 and IL-6 double blockade	IL-6	The combined blockade of CTLA-4 and IL-6 promotes the infiltration of CXCR3-expressing T cells by inducing CD4+ T cells to secrete IFN-γ.It can also alleviate immune-related adverse events.	(104)
	PD-L1 and IL-6 double blockade	IL-6	Combined blockade of IL-6 and PD-L1 inhibits pancreatic cancer progression and prolongs survival by increasing the infiltration of intratumoral effector CD8+ T cells and promoting the conversion of circulating CD4+ T cells to the Th1 phenotype.	(105)
	BAY11–7082 combined with IL-18	IL-18	Targeted inhibition of the NF-κB pathway using BAY11–7082 suppresses the promoting effects of IL-18 on tumor proliferation and invasion, enabling IL-18 to act as a co-stimulatory cytokine to facilitate immunotherapy.	(106)
Chemotherapy	Anakinra	IL-1β	Anakinra inhibits the NF-κB activity of CAFs by interrupting the tumor-matrix IL-1β-IRAK4 feedforward circuit, attenuating the pro-survival and chemoresistance effects of CAFs on PDAC cells	(107)
	Anakinra	IL-1α	Anakinra inhibits treatment resistance due to IL-6-dependent STAT3 activation in tumor cells by inhibiting IL-1R1 signaling on pancreatic stellate cell PSCs, thereby reducing IL-6 secretion by PSCs, and enhancing chemotherapy efficacy	(112)

### 4.1 The evaluation of the prognosis of PC

In the diagnosis of PC, existing biomarkers have certain limitations. Take CA19-9, which is universally used in clinical practice, for example. Its sensitivity and specificity for early detection of PC are limited. Therefore, the search for more reliable diagnostic markers is extremely urgent. Notably, although a single biomarker performs poorly in diagnosis, the combined detection of CA19-9, CEA, CA125, and CA242 is significantly more accurate than a single serum biomarker. Their sensitivity and specificity are as high as 90.4% and 93.8%, respectively (95). Currently, the role of inflammatory factors in the diagnosis of PC remains unclear. However, emerging studies have indicated that they are gradually demonstrating value in predicting disease prognosis.

S Mitsunaga et al. discovered that the serum levels of IL-6 and IL-1β can predict the therapeutic efficacy of Gemcitabine (GEM) and the prognosis of patients with advanced PC. They measured the levels of pro-inflammatory cytokines in the serum of advanced PC patients receiving single-agent GEM treatment. Multivariate analysis showed that high levels of IL-6 and IL-1β are poor prognostic factors for overall survival. Compared with patients with low levels of both IL-6 and IL-1β, patients with high levels of both IL-6 and IL-1β have significantly shorter overall survival and progression-free survival, a lower tumor control rate, and a reduced high-dose intensity of GEM (96).

Moreover, multiple studies have collectively demonstrated that the inflammatory factor IL-13 is of great significance in evaluating patient prognosis. Rachel F Gabitass et al. conducted a comprehensive analysis of circulating MDSC and Tregs in patients with pancreatic, esophageal, and gastric cancers. They found that the MDSC value is an independent prognostic factor for patients with pancreatic and esophagogastric cancers. For every one - unit increase in MDSC, the risk of death in patients increases by 22%. Meanwhile, when evaluating a series of plasma cytokines, they found that the cytokine IL-13 in the plasma of patients was significantly elevated, and this elevation was positively correlated with the MDSC level (60). This suggests that IL-13 may play an important role in the process of MDSC exerting its functions. The study by Mandruzzato S et al. also confirmed this view: among tumor-induced CD11b(+) splenocytes, IL-4Rα(+) cells produce large amounts of inhibitory IL-13 and IFN-γ, while IL-4Rα (-) cells do not constitutively secrete these cytokines and are non-inhibitory. The full inhibitory function of MDSC in tumor-conditioned mice requires the coordinated action of IL-13 and IFNα released by cells in an autocrine manner (97). Additionally, Formentin et al. detected high levels of IL-13 in pancreatic ductal carcinoma cells, while normal pancreatic cells did not contain IL-13. They also found that in some PC cell lines, IL-13 can induce dose - dependent cell growth, and this phenomenon can be inhibited in a dose-dependent manner by an IL-13 neutralizing antibody, indicating that IL-13 is an autocrine growth factor in PC (98).

### 4.2 Therapeutic strategies targeting inflammatory factors to remodel TME

The unique TME of PC is a primary contributor to treatment failure with conventional monotherapies. As previously described, its key features include: An immunosuppressive microenvironment, Dense desmoplastic stroma, Hypoperfusion. The complex network of stromal cells and signaling pathways during TME formation poses significant therapeutic challenges. However, inflammatory factors serve as pivotal mediators connecting tumor cells, stromal components, and signaling cascades. Thus, targeting core inflammatory factors represents a viable strategy to remodel or reverse the pro-fibrotic/immunosuppressive TME, thereby enhancing existing therapies.

#### 4.2.1 Targeting pro-fibrotic factors: reversing the stromal barrier

TGF-β is a central pro-fibrotic driver of stromal remodeling in PC. Inhibiting its signaling pathway effectively suppresses cancer-associated fibroblast (CAF) activation and remodels the dense extracellular matrix (ECM), improving drug delivery and alleviating immune suppression.

Early studies reported gabapentin’s ability to inhibit ketogenic acid production in CAFs (99). Paradoxically, gabapentin dose-dependently elevates TGF-β1 levels in cancer cells. Jin Zhang et al. demonstrated that combining pirfenidone with gabapentin synergistically inhibits PDAC growth and overcomes apoptosis resistance. They subsequently developed GEP—a coordination nanomedicine incorporating gabapentin, pirfenidone, and epigallocatechin gallate (EGCG). This agent remodels TME by altering CAF phenotypes, overcoming “gabapentin resistance” caused by TGF-β1 upregulation while significantly increasing intratumoral functional CD8^+^ T cell infiltration (100).

Notably, myofibroblast-like CAFs (myCAFs; α-SMA^+^FAPα^+^) have recently gained attention as TGF-β-driven architects of pro-tumorigenic TME (101). Ning Pang et al. introduced SB525334—a selective TGF-β receptor I inhibitor—as a pioneering agent preceding nanochemotherapy. It ablates TGF-β signaling without compensatory autocrine secretion, disrupting the CAF barrier while normalizing microvasculature and improving TME perfusion. This paves the way for enhanced tumor accumulation and delivery of docetaxel-loaded micelles, ultimately boosting antitumor efficacy (102).

#### 4.2.2 Blocking immunosuppressive factors: restoring anti-tumor immunity

IL-6 and IL-18 are key immunosuppressive factors maintaining PC’s immunosuppressive microenvironment by suppressing T cell function.

PC’s poor immunogenicity, scarce neoantigens, and profoundly immunosuppressive TME render single-agent immune checkpoint inhibitors (ICIs) clinically ineffective. Although IL-6 typically mediates immune defense, accumulating evidence reveals its dual role in PC: directly promoting tumor proliferation/survival while driving immune escape via T cell exclusion and functional impairment (103). Preclinical studies confirm that targeting IL-6/IL-6R reverses TME immunosuppression: IL-6 blockade combined with immunotherapy significantly enhances intratumoral T cell infiltration, dismantles immunosuppressive barriers, and restores anti-tumor immunity, thereby potentiating ICI efficacy and suppressing tumor progression in murine PC models (104, 105).

**Figure 4:**
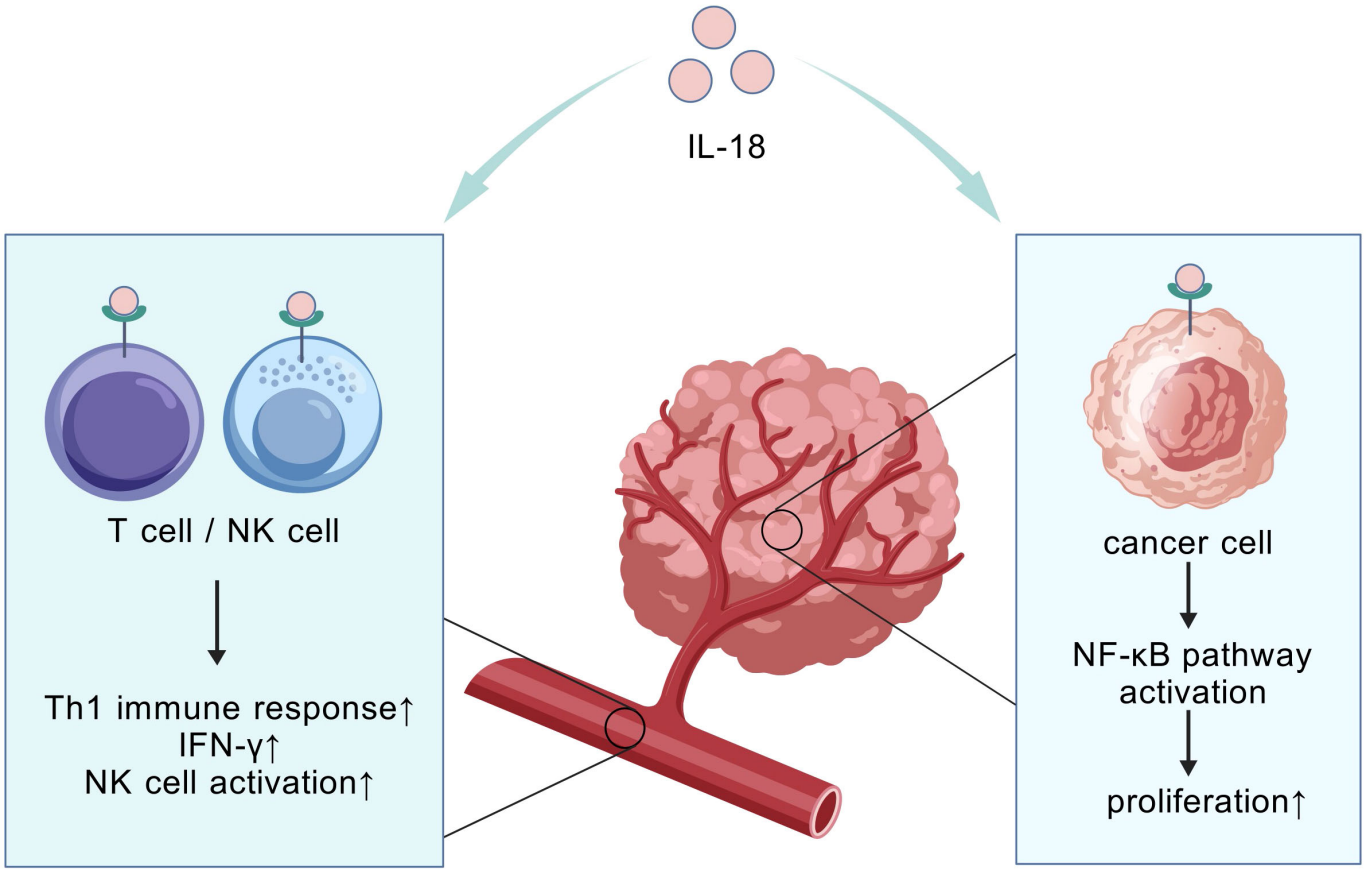
Compartment-specific IL-18 effects. The compartment-specific effect refers to the spatially dependent dual role of IL-18. It activates systemic anti-tumor immunity in circulation (correlating with improved survival), while locally within tumor tissue it promotes immunosuppression and tumor progression via NF-κB pathway activation.

Beyond cytokine blockade, a “function-selective modulation” strategy has been proposed to address the context-dependent duality of cytokines such as IL-18. This principle recognizes that cytokines may exert divergent biological effects in distinct microenvironments—a critical constraint for cytokine-based therapies. As demonstrated by Xingjun Guo et al., IL-18 exemplifies this duality in PC: systemic elevation correlates with anti-tumor immunity and prolonged patient survival, whereas high intratumoral levels activate NF-κB to promote invasion, metastasis, and reduced survival (**Figure 4**). Consequently, IL-18 monotherapy exhibits marginal efficacy. To resolve this, Guo et al. pioneered a novel co-targeting strategy combining IL-18 with NF-κB inhibitors. This approach selectively neutralizes IL-18’s pro-tumor effects within the TME while preserving its systemic immunostimulatory potential, significantly improving survival in murine PC models. This establishes IL-18/NF-κB co-targeting as a paradigm for overcoming context-dependent functional limitations of ambivalent cytokines (106).

#### 4.2.3 Modulating inflammatory signaling to sensitize chemotherapy

IL-1-mediated chemoresistance represents a major limitation for conventional therapies (e.g., gemcitabine). Targeting this inflammatory signaling hub disrupts resistance pathways and reverses TME-driven chemotherapy refractoriness.

Zhang et al. discovered that IRAK4 activation in CAFs promotes NF-κB-dependent IL-1β secretion, establishing a feedforward loop. Within this circuit, IL-1β further activates the IRAK4-NF-κB signaling pathway in both CAFs and PDAC cells. This pathway activation drives CAFs to secrete excessive profibrotic factors (e.g., collagen type I), leading to the formation of a dense, fibrotic stroma. Critically, this fibrotic stroma exerts dual barrier effects: it physically impedes the penetration of chemotherapeutic agents (e.g., gemcitabine) while simultaneously creating a protective, pro-survival niche for PDAC cells. In preclinical studies, combining the IRAK4 inhibitor AS2444697 or IL-1β-neutralizing antibodies with gemcitabine significantly suppressed tumor growth and reduced fibrosis, highlighting the therapeutic potential of disrupting this signaling loop (107).

Separately, constitutive activation of the pro-inflammatory STAT3 pathway is a key biomarker of PDAC chemoresistance. PSCs release IL-6 to cross-talk with tumor cells, activating STAT3 signaling and promoting invasive phenotypes. STAT3 inhibition suppresses PDAC growth/invasion and profoundly remodels stroma to improve drug delivery and therapeutic response (108–111). Austin R. Dosch et al. further identified tumor-derived IL-1α as an upstream mediator of PSC-driven IL-6 release and STAT3 activation in TME. Consequently, IL-1R1 inhibitor anakinra partially overcomes STAT3-mediated chemoresistance in PDAC (112).

Collectively, these findings position anakinra combined with chemotherapy as a promising strategy to counteract IL-1α/β-driven resistance mechanisms.

## 5 Conclusion

With the deepening of research in recent years, the understanding of tumors has expanded from focusing solely on tumor cells to also considering the complex regulatory roles of the TME. In the TME, inflammatory factors play a key role in intercellular communication, directly or indirectly interacting with tumor cells and exerting significant regulatory effects on tumor progression. This review highlights several important factors in PC TME research in recent years, such as IL-1β, IL-4, IL-13, IL-15, IL-17, IL-18, and TNF-α. These factors influence the phenotypes of cells in the microenvironment through pathways such as MAPK, PI3K-Akt, JAK-STAT, and NF-κB. leading to changes such as an immunosuppressive TME, dense desmoplastic stroma, and hypoperfusion. These changes are critical nodes in the development and progression of PC and represent three distinct characteristics of the PC microenvironment compared to other tumors. They are closely associated with poor prognosis and treatment resistance in PC (**Table 2**).

**Table 2:** Summary table: key inflammatory mediators in pancreatic cancer.

Mediators	Signaling pathways	Core effects	Therapeutic strategies	Ref.
IL-1β	CAFs → IRAK4 → NF-κB → pro-fibrotic factors	Promotes collagen secretion by CAFs (fibrosis), impeding chemotherapy delivery	Targeting IRAK4 (inhibitor AS2444697)	(54, 107)
	CAFs/PDAC cells → IL-1β-IRAK4-NF-κB loop → IL-1β amplification	Enhances PDAC cell proliferation and chemoresistance	IL-1β-neutralizing antibodies (in combination with gemcitabine)	
	TAMs → PGE_2_/TNF → IL-1β secretion	Drives immune evasion (TME immunosuppression)		
IL-4	Pancreatic cancer cells → IL-4/IL-4R → IRS-MAPK/Akt/Stat3 → proliferation	Directly promotes cancer cell proliferation	IL-4-neutralizing antibodies	(57–59)
	Macrophages → IL-4/IL-4R → JAK-STAT6 → M2 polarization	Establishes immunosuppressive TME (M2 macrophages, T cell inactivation)		
	T cells → IL-4/IL-4R → STAT6 → T cell dysfunction		CAR-T cell engineering (expressing IL-4-based inverted cytokine receptors)	
IL-13	PSCs → IL-13/IL-13Rα → M2 macrophage polarization	Facilitates tumor progression (associated with poor prognosis)	IL-13Rα2 (CAR-T targeting, tumor-specific)	(65, 66)
	Macrophages → IL-13/IL-13Rα1 → Ym1+ macrophages → fibrosis	Supports fibrosis and pre-neoplastic lesions (ADM/panIN)		
IL-15	CD8^+^ T/NK cells → IL-15/IL-15Rα → JAK-STAT5 → enhanced cytotoxicity	Enhances anti-tumor immunity (proliferation and cytotoxicity of CD8^+^ T/NK cells)	IL-15 super-agonist (Niz985, mimicking exercise-induced immune activation)	(68)
	CGRP → CAFs → IL-15↓ → NK cell dysfunction	Mediates exercise-induced anti-tumor effects		
IL-17	CAFs → IL-17A/IL-17RA → TNF/NF-κB → iCAF	Drives CAF differentiation (iCAFs)	Targeting IL-17B/IL-17RB axis (blocking metastasis-related chemokine secretion)	(72, 74)
	Cancer cells → IL-17B/IL-17RB → ERK1/2 → chemokine induction	Inhibits CD8^+^ T cell infiltration		
	Neutrophils→IL-17→PADI4-dependent histone citrullination → NET formation			
IL-18	Macrophages → IL-18/IL-18R → NF-κB/STAT3 → M2 polarization	Promotes M2 macrophage infiltration (immunosuppression)	Combination with NF-κB inhibitors (abrogating pro-tumor effects)	(76–78)
	CD8^+^ T cells → IL-18/IL-18R → IL-2/STAT5 + AKT/mTOR → T cell exhaustion	Drives CD8^+^ T cell exhaustion (immune evasion)	Targeting IL-18/IL-18R (blocking T cell exhaustion)	
	IL-18 → eosinophils → TGF-β/SMAD4 → fibrosis	Induces fibrosis	Targeting IL-18/IL-18R	
TNF-α	DCs → TNF-α/TNFR1 → immune suppression→ tumor immune escape	Inhibits DC activation (immunosuppression)	Blocking TNFR1 (restoring DC function)	(81, 84, 86)
	Cancer cells/CAFs →TNF-α/TNFR1 → NF-κB → cell survival	Drives CAF differentiation, promoting fibrosis and chemoresistance		
	Neutrophils & cancer cells → tmTNF/TNFR2 → NF-κB/STAT3 →CXCL1/IL-6 feedforward loop		Targeting neutrophil-tmTNF-TNFR2 interaction	
PGE_2_ (key subtype)	DCs → PGE_2_/EP2/EP4 →cAMP/PKA → XBP1s/ER Stress + ROS/4-HNE↑→ DC Dysfunction	Inhibits DC activation	Targeting COX-2 (reducing PGE_2_ synthesis)	(91–94)
	Cancer cells → PGE_2_ → FGF1 → CAFs → FGFR-MAPK → VEGFA	Promotes fibrosis (collagen secretion) and angiogenesis (VEGFA)		
	T cells → PGE_2_/EP2/EP4 → PKA/PI3K → T cell suppression	Suppresses T cell (including CAR-T) proliferation and anti-tumor function	Blocking EP2/EP4 receptors (enhancing CAR-T efficacy)	

Clinically, inflammatory factors serve as potential biomarkers and therapeutic targets. Treatments such as nanotechnology, immunotherapy (e.g., IL-6 blockade with anti-CTLA-4), and chemotherapy (using agents like IL-1β antagonists) are being explored. However, challenges persist. The mechanisms of inflammatory factors are not fully understood, and their complex effects hinder therapy development. Future research should focus on deepening our understanding through molecular, cellular, and *in vivo* studies to enable personalized treatments.
